# Comprehensive genome analysis of two novel *Saccharopolyspora* strains—*Saccharopolyspora montiporae* sp. nov. and *Saccharopolyspora galaxeae* sp. nov. isolated from stony corals in Hainan

**DOI:** 10.3389/fmicb.2024.1432042

**Published:** 2024-11-13

**Authors:** Yuhui Xie, Fenfa Li, Qingyi Xie, Fandong Kong, Yun Xu, Qingyun Ma, Wenqiang Wu, Dongyi Huang, Xinqiang Xie, Shuangqing Zhou, Youxing Zhao, Xiaolong Huang

**Affiliations:** ^1^School of Life and Health Sciences, Hainan University, Haikou, China; ^2^Frontiers Science Center for Synthetic Biology and Key Laboratory of Systems Bioengineering (Ministry of Education), School of Chemical Engineering and Technology, Tianjin University, Tianjin, China; ^3^Haikou Key Laboratory for Research and Utilization of Tropical Natural Products, Institute of Tropical Bioscience and Biotechnology, Chinese Academy of Tropical Agricultural Sciences, Haikou, China; ^4^Guangdong Institute of Microbiology, Guangdong Academy of Sciences, Guangzhou, China; ^5^College of Pharmacy, Guilin Medical University, Guilin, China

**Keywords:** marine actinobacterium, *Saccharopolyspora*, polyphasic taxonomy, comparative genomics, coral commensal microbes

## Abstract

Marine actinomycetes exhibit a high level of biodiversity and possess significant potential for the production of high-value secondary metabolites. During the course of investigation of marine actinobacteria from corals, two *Saccharopolyspora* strains, namely, HNM0983^T^ and HNM0986^T^, were isolated from stony corals collected from the coastal area of Hainan Island. The 16S ribosomal RNA (rRNA) gene sequence analysis revealed that these two strains are putative novel taxa of the genus *Saccharopolyspora*. Whole-genome sequencing comparisons further confirmed the two strains as belonging to two novel *Saccharopolyspora* species, which can be distinguished phenotypically and chemically from their current closest phylogenetic relatives. Some genomic information of the genus *Saccharopolyspora* was compared for evaluating the production capacity of secondary metabolites. A total of 519 biosynthetic gene clusters (BGCs) from the genus *Saccharopolyspora* were used for analysis, and terpene BGCs were found to be widespread and most abundant in this genus. In addition, abundant novel BGCs in the genus *Saccharopolyspora* are not clustered with the known BGCs in the database, indicating that the metabolites of the genus *Saccharopolyspora* deserve further exploration. On the basis of these presented results, *Saccharopolyspora* montiporae sp. nov. (type strain = HNM0983^T^ = CCTCC AA 2020014^T^ = KCTC 49526^T^) and *Saccharopolyspora galaxeae* sp. nov. (type strain = HNM0986^T^ = CCTCC AA 2020011^T^ = KCTC 49524^T^) are proposed as the names for the new strains, respectively.

## 1 Introduction

The genus *Saccharopolyspora*, a member of the family *Pseudonocardiaceae*, was first proposed in 1975 (Lacey and Goodfellow, 1975) and revised in 1989 (Korn-Wendisch et al., 1989). The genus *Saccharopolyspora* currently comprises 39 species with validly published and correct names^1^ (Sayed et al., 2020). The genus *Saccharopolyspora* has a wide distribution and is often found in some extreme environments, such as mangrove sediments (Suksaard et al., 2018), marine invertebrates (Pimentel-Elardo et al., 2008; Souza et al., 2017), deep-sea sediments (Jiang et al., 2016), hypersaline lakes (Lv et al., 2014; Xia et al., 2017), and deserts (Yang et al., 2018; Saygin et al., 2021).

The genus *Saccharopolyspora* has a high application potential and needs further exploration, especially in the field of novel or active natural product discovery. Some crucial drugs, agricultural antibiotics, and active compounds have been found in the genus *Saccharopolyspora*, such as erythromycin A, spinosyn A and D (Kim and Goodfellow, 2015), cebulantin (Moon et al., 2019), and compound KR21-0001A (Janthanom et al., 2024). More importantly, the majority of members of the genus are regarded as valuable sources of many secondary metabolites with diverse chemical classes, such as glycolipids, alkaloids, macrolides, peptides, oligosaccharides, and quinones, which possess different biological activities, including cytotoxic, insecticidal, and antimicrobial (Sayed et al., 2020). Recent genome-wide comparative analysis results further support the metabolic potential of *Saccharopolyspora* taxa (Saygin et al., 2021).

As a common marine organism, coral is widely distributed in the ocean. Corals provide a relatively stable living environment for marine microorganisms, and a large number of novel microorganisms have been isolated from corals in recent years, such as *Saccharopolyspora coralli* (Zhou et al., 2020), *Nocardiopsis coralli* (Li et al., 2021), and *Alkalimarinus coralli* (Li et al., 2023). In this study, strains HNM0983^T^ and HNM0986^T^ were isolated from the stony corals *Montipora foliosa* and *Galaxea astreata*, respectively, which were collected from the sea of Wenchang, Hainan, China. These two strains were identified as two novel species of the genus *Saccharopolyspora* by polyphasic analysis. In addition, we compared their genomic differences with other *Saccharopolyspora* strains. Comprehensive genome analyses showed that strains HNM0983^T^ and HNM0986^T^ have the potential for novel bioactive compounds.

## 2 Materials and methods

### 2.1 Isolation and maintenance of actinobacteria

The coral samples were collected from the coast of the eastern suburbs of Wenchang City, Hainan Province, China (110.88 E, 19.53 N). The collected coral samples were immediately placed in sterile sample bags and stored in the freezer (−80°C). The Coral Reef Protection Laboratory, School of Oceanography, Hainan University completed coral identification and classification. Coral samples were ground into powder and diluted with sterile seawater. The diluted coral homogenate was spread on Gause’s synthetic medium, which was prepared using 50% (v/v) seawater and supplemented with 75 mg of potassium dichromate per liter (Gao et al., 2012). All plates were incubated at 28°C for 4 weeks, and single colonies of actinomycetes were purified continuously on ATCC 172 medium (Hong et al., 2009). Purified cultures were stored in 20% (v/v) glycerol and frozen at −80°C.

### 2.2 Phylogenetic analyses

PCR amplification of the 16S rRNA sequence was described in a previous study (Huang et al., 2012). Almost complete 16S rRNA sequences from strains HNM0983^T^ (1,464 bp) and HNM0986^T^ (1,502 bp) were compared with the database on the EzBioCloud website (Yoon et al., 2017a). The 16S rRNA phylogenetic tree was constructed using the neighbor-joining method (Saitou and Nei, 1987), the maximum-likelihood method (Felsenstein, 1981), and the maximum-parsimony method (Fitch, 1981) in MEGA 7.0 software, respectively (Kumar et al., 2016). The topologies of the 16S rRNA phylogenetic trees were evaluated with 1,000 iterations for bootstrap support (Felsenstein, 1985). The genomic DNA was extracted by using the Wizard^®^ Genomic DNA Purification Kit (Promega Beijing Biotech Co., Ltd). All sequencing services were performed by Shanghai Majorbio Biopharm Technology Co., Ltd. (Shanghai, China). The Illumina HiSeq X Ten was used as a sequencing platform and assembled by SOAPdenovo (version 2.04; Luo et al., 2012). Genome quality assessment, genome circle map construction, and genome annotation were performed by the Bacterial and Viral Bioinformatics Resource Center (BV-BRC) server (Olson et al., 2023). The whole-genome phylogenomic tree was constructed on the Type Strain Genome Server (TYGS; Meier-Kolthoff and Göker, 2019).

### 2.3 Morphological and physiological properties

ISP 1-ISP 7 agar (Shirling and Gottlieb, 1966) and Czapek agar (Kinderlerer, 1995) were used to test the state of growth, with all culture mediums supplemented with 16.5 g/L sea salt and cultured at 28°C for 3 weeks. The designation of colony color was determined by comparison with the American Inter-Society Color Council–National Bureau of Standards (ISCC–NBS) color charts (Kelly, 1964). NaCl tolerance (concentrations ranged from 0 to 25% with 1% intervals), pH tolerance (3.0–11.0, in intervals of 1.0 pH units), and growth temperature range (0°C, 4°C, 10°C, 28°C, 37°C, 40°C, and 45°C) were tested on International Streptomyces Project-2 (ISP 2) medium. Carbon and nitrogen source utilization tests were consistent with previous reports (Xie et al., 2020; Williams et al., 1983). Hydrolysis of starch, production of H_2_S and melanin, milk coagulation, and nitrate reduction were carried out as described previously (Goodfellow, 1971; Athalye et al., 1985a). To ensure the accuracy of the test results, the above tests were repeated three times.

### 2.4 Chemotaxonomic characteristics

For chemotaxonomic analyses, strains HNM0983^T^ and HNM0986^T^ were cultured in ATCC172 broth at 28°C for 7 days. Whole-cell hydrolysate determination was performed according to previous reports (Lechevalier and Lechevalier, 1970). Polar lipids were extracted and examined according to published procedures (Minnikin et al., 1984). Menaquinones were analyzed using high-performance liquid chromatography (HPLC; Collins et al., 1977; Tamaoka et al., 1983). Extraction and analysis of cellular fatty acids were performed by using the method of Athalye et al. (1985b).

### 2.5 Comparative genome analyses

The digital DNA–DNA hybridization (dDDH) analysis was performed using the DSMZ (German Collection of Microorganisms and Cell Cultures) Genome-to-Genome Distance Calculator platform (Meier-Kolthoff et al., 2013). Average nucleotide identity (ANI) values of the genomes of strains with closely related type strains were calculated using the ANI Calculator (Yoon et al., 2017b). Pan-genome analysis was performed using the Pan-Genome Analysis Web Server (PGAP; Chen et al., 2018). Protein clustering analysis was performed using OrthoVenn2 (Xu et al., 2019). Visual comparisons of the whole genomes of strains HNM0983^T^ and HNM0986^T^ and other most closely related type strains were performed using the BV-BRC server (Olson et al., 2023). The biosynthetic gene clusters (BGCs) were annotated using antiSMASH 7.0 (Blin et al., 2023). The similarity network of BGCs was constructed using BiG-SCAPE version 1.1.2, employing the cutoff threshold of 0.6 (Navarro-Muñoz et al., 2020), and visualized using Cytoscape version 3.10.2 (Otasek et al., 2019).

## 3 Results and discussion

### 3.1 Phylogenetic analysis

**Figure 1:**
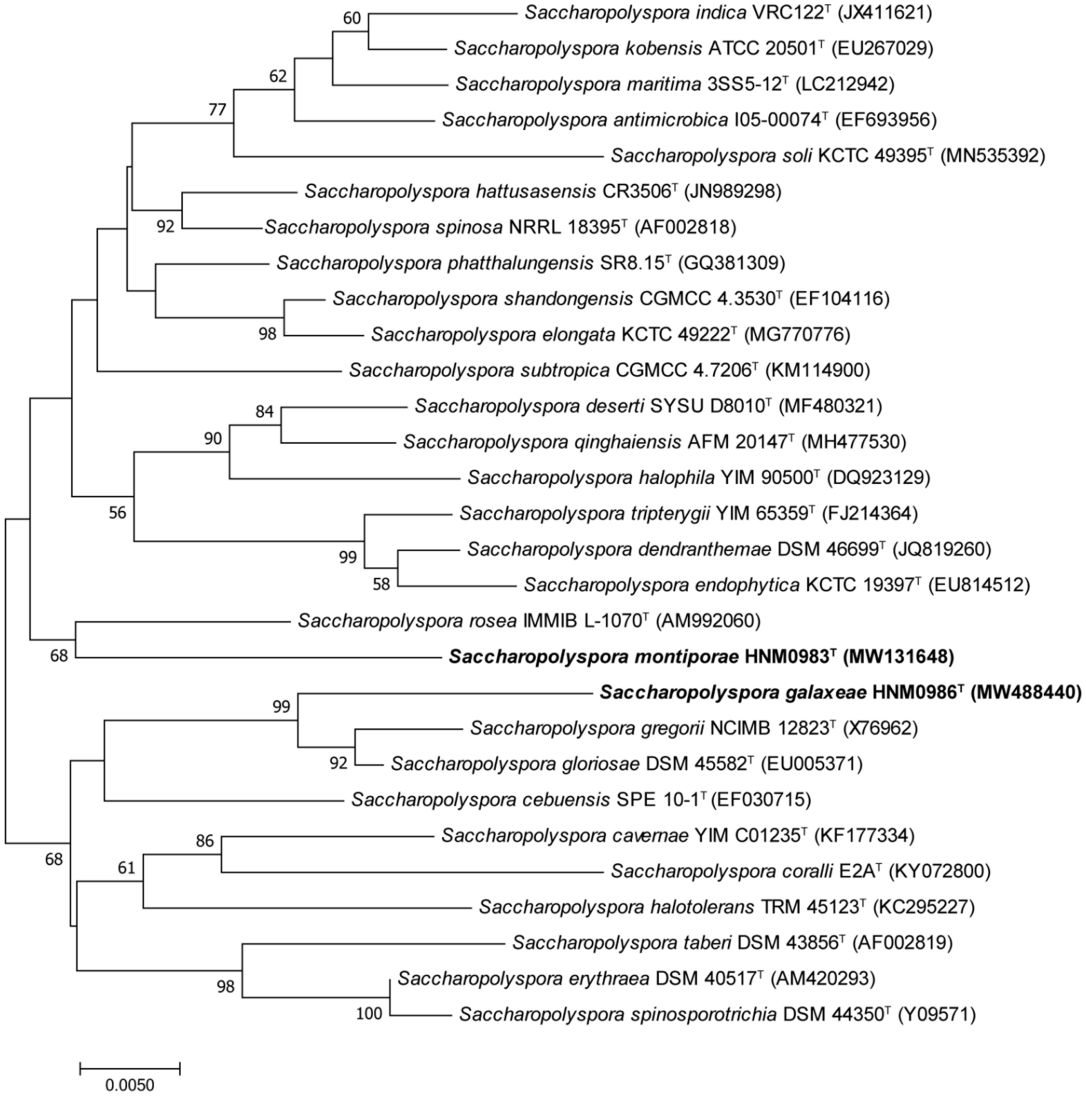
Neighbor-joining phylogenetic tree derived from 16S rRNA sequences, showing the relationships between HNM0983^T^, HNM0986^T^, and other type strains of the genus *Saccharopolyspora*. Only values above 50% are shown. Bar indicates 5 nucleotide substitutions per 1000 nucleotides.

**Figure 2:**
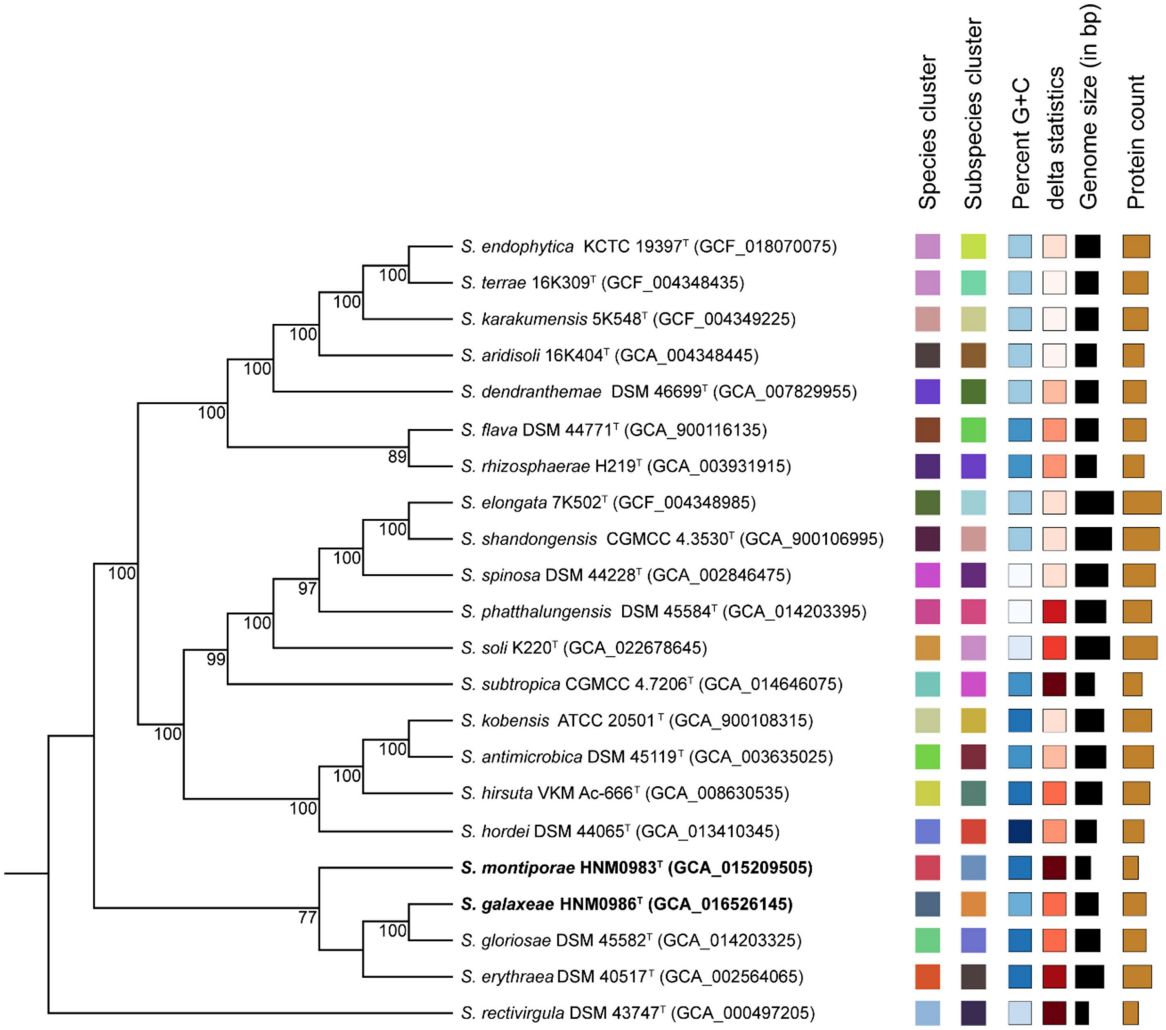
Genomic phylogenetic tree constructed by TYGS. Tree inferred with FastME 2.1.6.1 from Genome-Basic Local Alignment Search Tool (BLAST) Distance Phylogeny (GBDP) distances calculated from genome sequences. The branch lengths are scaled according to the GBDP distance formula d5. The numbers above branches are GBDP pseudo-bootstrap support values >60% from 100 replications, with an average branch support of 84.6%. Leaf labels with different colors indicate species and subspecies clusters.

Strain HNM0983^T^ had the highest 16S rRNA similarity with *S. rosea* IMMIB L-1070^T^ (96.80%), *Saccharopolyspora spinosa* NRRL 18395^T^ (96.75%), *Saccharopolyspora phatthalungensis* SR8.15^T^ (96.75%), and *Saccharopolyspora hattusasensis* CR3506^T^ (96.41%). Strain HNM0986^T^ had the highest 16S rRNA similarity with *Saccharopolyspora gloriosae* DSM 45582^T^ (97.85%), *Saccharopolyspora gregorii* NCIMB 12823^T^ (97.70%), *S. cebuensis* SPE 10-1^T^ (96.05%), and *S. rosea* IMMIB L-1070^T^ (96.02%). Obviously, all of the aforementioned similarities are lower than the threshold suggested for species demarcation (98.7%; Chun et al., 2018). In the 16S rRNA phylogenetic tree (constructed by the neighbor-joining method), strain HNM0983^T^ and *S. rosea* IMMIB L-1070^T^ formed a distinct monophyletic lineage, while strain HNM0986^T^ formed another distinct monophyletic lineage with *S. gloriosae* DSM 45582^T^ and *S. gregorii* NCIMB 12823^T^ (Figure 1). Similar 16S rRNA phylogenetic analysis results also appeared in the maximum-likelihood (Supplementary Figure S1) and maximum-parsimony (Supplementary Figure S2 phylogenetic trees). Obviously, based on the result of 16S rRNA phylogenies, strains HNM0983^T^ and HNM0986^T^ were found to belong to the genus *Saccharopolyspora*, and they were all different from the known type strains. Furthermore, the phylogenetic tree constructed by whole-genome sequences also obtained similar results. Based on the available genomic information for the genus *Saccharopolyspora* (a total of 22 type strains), strains HNM0983^T^, HNM0986^T^, *S. gloriosae* DSM 45582^T^, and *S. erythraea* DSM 40517^T^ were most closely related at the genomic level (Figure 2). Overall genomic relatedness indices (OGRI) were used to further determine the relationship among all these *Saccharopolyspora* strains.

For strain HNM0983^T^, dDDH values compared with other strains ranged from 20.4% (*Saccharopolyspora rectivirgula* DSM 43747^T^) to 22.8% (*Saccharopolyspora antimicrobica* DSM 45119^T^; Supplementary Table S1), which were significantly lower than the recommended thresholds of 70% for species demarcation (Goris et al., 2007). Similarly, ANI values varied from 77.2% (*Saccharopolyspora aridisoli* 16K404^T^, *Saccharopolyspora dendranthemae* DSM 46699^T^, and *Saccharopolyspora rhizosphaerae* H219^T^) to 79.0% (*Saccharopolyspora hirsuta* VKM Ac-666^T^; Supplementary Table S1), remaining well below the recommended cutoff value of 95.0–96.0% for species recognition (Richter and Rosselló-Móra, 2009). For strain HNM0986^T^, dDDH values ranged from 21.2% (*S. dendranthemae* DSM 46699^T^) to 24.6% (*S. gloriosae* DSM 45582^T^; Supplementary Table S1), and ANI values ranged from 76.6% (*S. rectivirgula* DSM 43747^T^) to 81.3% (*S. gloriosae* DSM 45582^T^). Independent dDDH and ANI tests confirmed that strains HNM0983^T^ and HNM0986^T^ are distinct from existing type strains. Consequently, strains HNM0983^T^ and HNM0986^T^ represent novel species of the genus *Saccharopolyspora*.

### 3.2 Morphological and physiological characteristics

The novel strains HNM0983^T^ and HNM0986^T^ showed different growth states on a variety of media, but neither was able to grow on ISP 4 agar. The growth status of HNM0983^T^ was superior to that of HNM0986^T^ on the majority of the tested media, and the growth differences between the two strains are detailed in Supplementary Table S2. The optimal NaCl concentration of strain HNM0983^T^ was 0–1% (w/v), but the maximum NaCl tolerance was 20%, the pH tolerance range was 5.0–10.0, and the optimal growth pH was 8.0–9.0. The growth temperature of HNM0983^T^ ranged from 10°C to 37°C, and the optimal temperature was 28°C. Strain HNM0986^T^ had the same growth temperature range as HNM0983^T^ but was different in NaCl concentration tolerance and pH tolerance. The optimal NaCl concentration of strain HNM0986^T^ was 0–7% (w/v), but the maximum NaCl tolerance was 15%, the pH tolerance range was 5.0–10.0, and the optimal growth pH was 7.0. In addition, there were some differences in the utilization of single carbon and nitrogen sources between these two strains, such as sucrose, raffinose, maltose, cellobiose, D-galactose, D-sorbitol, D-mannose, D-melibiose, L-asparagine, and L-arginine. Strains HNM0983^T^ and HNM0986^T^ can also be distinguished from their phylogenetic neighbors by biochemical reactions and degradability tests, the results of which are shown in Table 1.

**Table 1:** Comparison of physiological properties and chemotaxonomic characteristics between two new strains and their closely related strains.

Characteristics	1	2	3^a^	4^a^	5^a^
Single carbon source utilization					
Sucrose	−	+	+	−	+
Raffinose	−	+	−	−	+
Maltose	−	+	+	+	+
Cellobiose	−	+	−	+	ND
Trehalose	−	−	+	−	+
L-rhamnose	−	−	−	+	+
L-arabinose	−	−	+	+	+
D-glucose	+	+	+	+	+
D-fructose	+	+	+	+	+
D-galactose	+	−	+	−	+
D-xylose	−	−	+	+	+
D-sorbitol	+	−	+	ND	ND
D-mannose	−	+	+	+	+
D-melibiose	−	+	ND	ND	ND
Single nitrogen source utilization					
L-asparagine	+	−	ND	+	ND
L-serine	−	−	ND	+	−
L-histidine	+	+	ND	+	ND
L-arginine	+	−	ND	+	ND
L-adenine	+	+	ND	+	ND
L-proline	−	−	ND	+	+
L-cysteine	+	+	ND	ND	ND
Physiological and biochemical characteristics					
Production of H_2_S	+	+	ND	ND	ND
Tyrosine hydrolysis	−	−	+	+	+
Nitrate reduction	+	+	−	−	ND
Milk coagulation	−	+	ND	ND	ND
Amylase	−	−	ND	ND	ND
Melanin	+	−	−	−	ND
Growth Temperature	10–37°C	10–37°C	22–42°C	10–32°C	10–35°C
pH	5.0–10.0	5.0–10.0	6.0–8.0	6.0–8.0	ND
NaCl tolerance (%)	0–20	0–15	ND	0–11	ND
Chemotaxonomic characteristics					
Major fatty acids	*iso*-C_15:0_ (16.18%), *anteiso*-C_15: 0_ (12.77%), *iso*-C_16:0_ (17.95%), *iso*-C_17:0_ (7.98%), *anteiso*-C_17: 0_ (27.18%)	*iso*-C_15: 0_ (19.31%), *iso*-C_16: 0_ (29.17%), *anteiso*-C_17: 0_ (24.41%)	*iso*-C_16:0_, *iso*-C_17:0_, *anteiso*-C_17: 0_	*iso*-C_16:0_, *anteiso*-C_17: 0_, C_17:1_ *cis9*	C_16: 0_, C_17: 0_
Major menaquinones	MK-9(H_4_)	MK-9(H_4_)	MK-9(H_4_)	MK-9(H_4_)	MK-9(H_4_)
Major polar lipids	PG PI PIM PC PE PL NPG	PG PI PC PE DPG	DPG PG PC PI	PC PE PME DPG PG PI PIM	DPG PI PC PG
Genome feature					
Genome size	4.16 Mbp	6.39 Mbp	ND	6.83 Mbp	ND
DNA G + C content	71.4%	70.3%	ND	71.6%	74%

Strains: 1, HNM0983^T^; 2, HNM0986^T^; 3, *S. rosea* IMMIB L-1070^T^; 4, *S. gloriosae* DSM 45582^T^; 5, *S. gregorii* NCIMB 12823^T^. +, Positive; −, negative; ND, not determined. PG, phosphatidylglycerol; PI, phosphatidylinositol; PIM, phosphatidylinositol mannosides; PC, phosphatidylcholine; PE, phosphatidylethanolamine; PL, unknown phospholipid; NPG, phosphatidyl-N-acetylglucosamine; DPG, diphosphatidylglycerol; PME, phosphatidyl methylethanolamine.

a Data for reference strains were taken from Kim and Goodfellow (2015), Yassin (2009), Qin et al. (2010), Goodfellow et al. (1989), and the National Center for Biotechnology Information (NCBI).

### 3.3 Chemotaxonomic analyses

The chemical taxonomic analyses of strains HNM0983^T^ and HNM0986^T^ showed that they had characteristics of the genus *Saccharopolyspora* (Kim and Goodfellow, 2015). They all contained MK-9(H_4_) as the major menaquinone and meso-diaminopimelic acid as the diagnostic diamino acid. The main differences in the chemical classification of the genus *Saccharopolyspora* were focused on the composition of fatty acids and polar lipids. The fatty acid composition of HNM0983^T^, HNM0986^T^, and closely related strain species is shown in Table 1. *Iso*-C_15:0_ is a unique fatty acid component, only detected in HNM0983^T^ and HNM0986^T^. The major polar lipids of strain HNM0983^T^ contained phosphatidylglycerol, phosphatidylinositol, phosphatidylinositol mannosides, phosphatidylcholine, phosphatidylethanolamine, phosphatidyl-N-acetylglucosamine, and some unknown phospholipids (Supplementary Figure S3). The major polar lipids of strain HNM0986^T^ were composed of phosphatidylinositol, phosphatidylcholine, phosphatidylglycerol, phosphatidylethanolamine, and diphosphatidylglycerol (Supplementary Figure S4). Phosphatidylcholine and phosphatidylglycerol were detected in all of the closely related strains, while diphosphatidylglycerol was detected in all closely related strains except strain HNM0983^T^ (Table 1). In summary, HNM0983^T^ and HNM0986^T^ can be differentiated from closely related strains within the genus *Saccharopolyspora* through chemical classification.

### 3.4 Genome feature and comparative genome analyses

**Figure 3:**
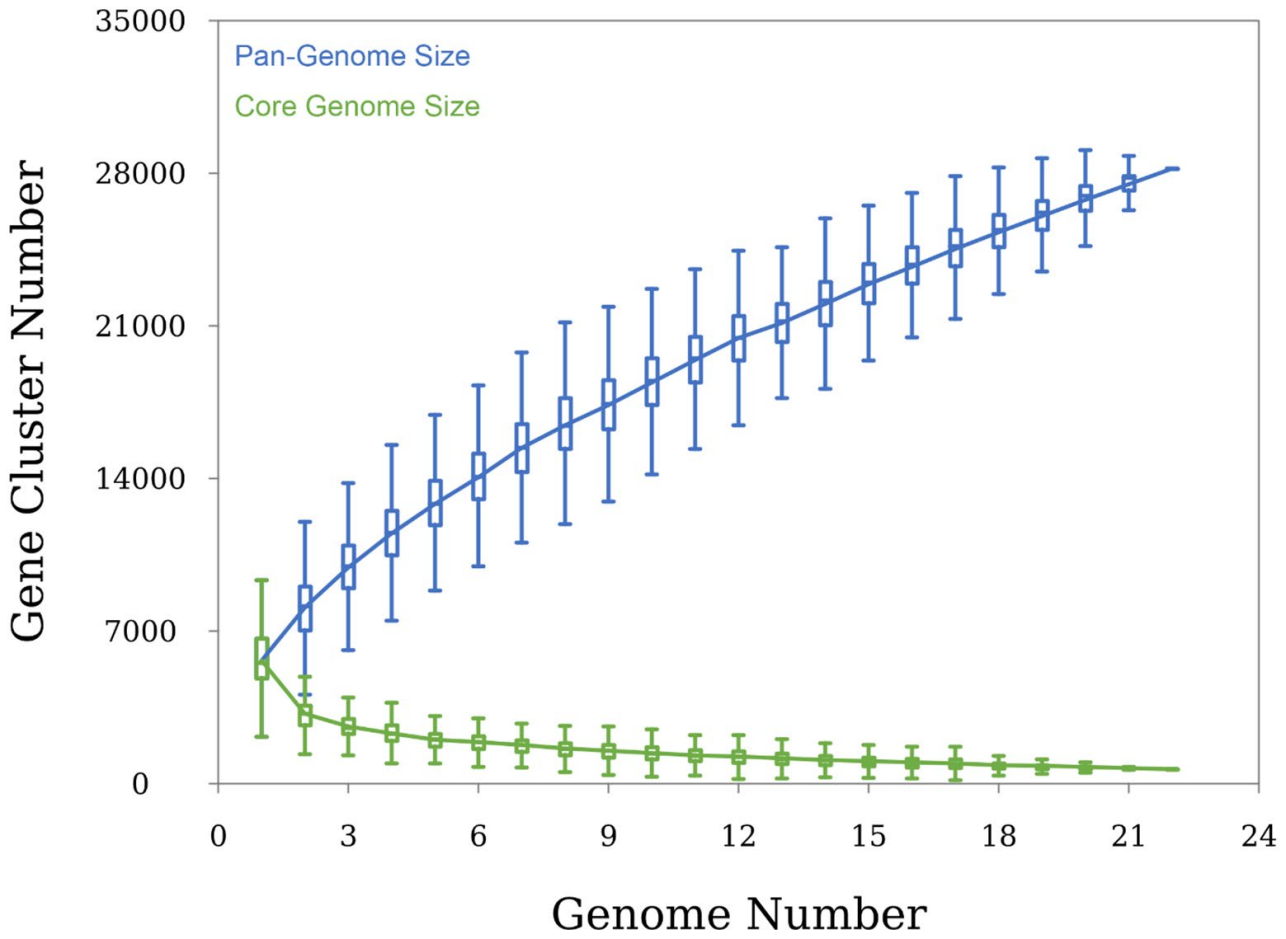
Pan-genome analysis of the genus *Saccharopolyspora* (conducted on 22 type strains of the genus *Saccharopolyspora*).

**Figure 4:**
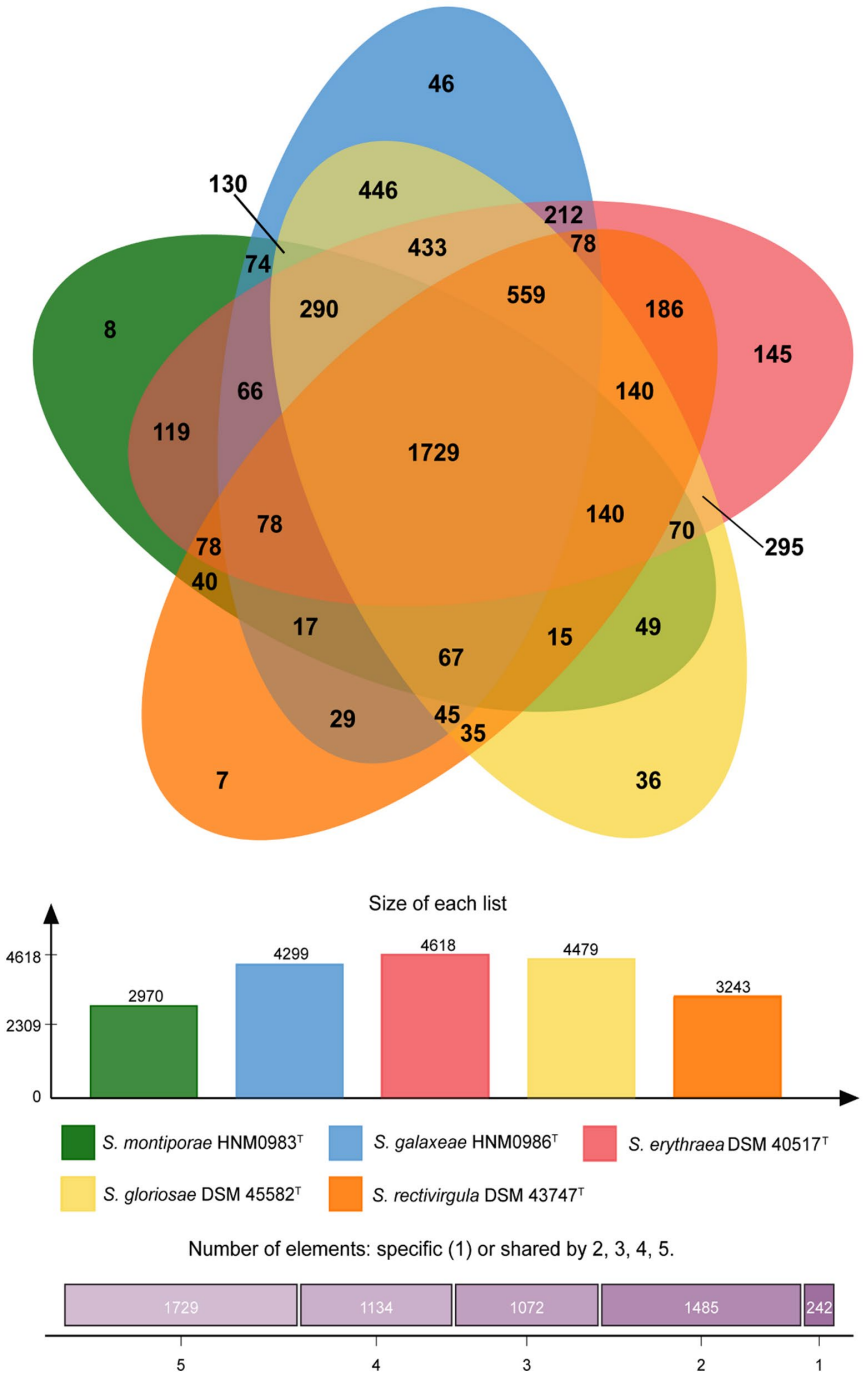
Protein cluster analysis of strains HNM0983^T^, HNM0986^T^, and three closely related strains of *Saccharopolyspora*.

**Figure 5:**
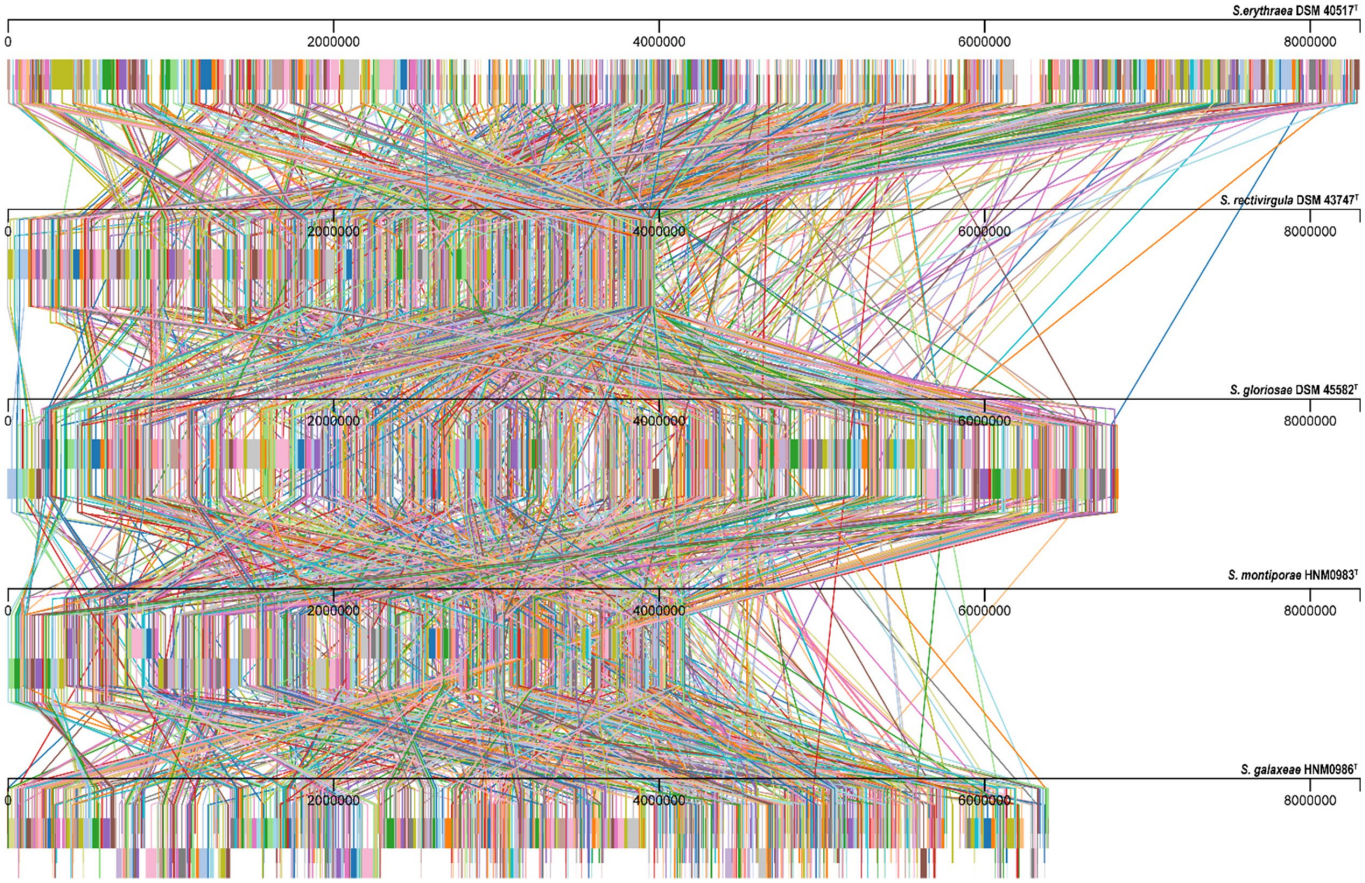
Alignment results of genome homologous regions of strains closely related to strains HNM0983^T^ and HNM0986^T^. Homologous regions are connected by lines.

Almost complete genomes of strains HNM0983^T^ and HNM0986^T^ were deposited in GenBank (the accession numbers JADEYC000000000 and JADDUE000000000, respectively). The sizes of the genomes of strains HNM0983^T^ and HNM0986^T^ were 4.16 and 6.39 Mbp, respectively. The G + C contents of strains HNM0983^T^ and HNM0986^T^ were 71.4 and 70.3%, respectively. The genome quality assessment and genome circular maps are shown in Supplementary Figure S5. The pan-genome analysis showed that with the addition of new genomes, the size of the pan-genome was further increased, indicating that the structure of this pan-genome is open type (Figure 3). In the open type of pan-genome, strains may contain some unique genes, so we compared the genomic composition of strains closely related to HNM0983^T^ and HNM0986^T^. The clustering of proteins of five related strains is shown in Figure 4. A total of 1729 homologous proteins were annotated in these strains, and each strain also contained some unique protein-coding genes. It is worth noting that based on the visual genome map, a large number of gene rearrangements occur in the genus *Saccharopolyspora* (Figure 5), even in closely related strains, which is consistent with the previous report (Saygin et al., 2021).

The marine environment has unfavorable factors such as high salinity, low temperature, and oligotrophic nutrition. Marine microorganisms may have some special metabolic pathways to enhance environmental resistance and interspecific competitiveness (Chen et al., 2022). The genome annotation of strains HNM0983^T^ and HNM0986^T^ showed a large number of genes related to habitat adaptation, such as heat shock proteins, cold shock proteins, betaine production, Na^+^ transport, heavy metal resistance, and transport (Supplementary Tables S3, S4). These genes provide important support for the homeostasis of the strain itself. In addition, secondary metabolites may also be important defensive weapons against potential threats.

**Figure 6:**
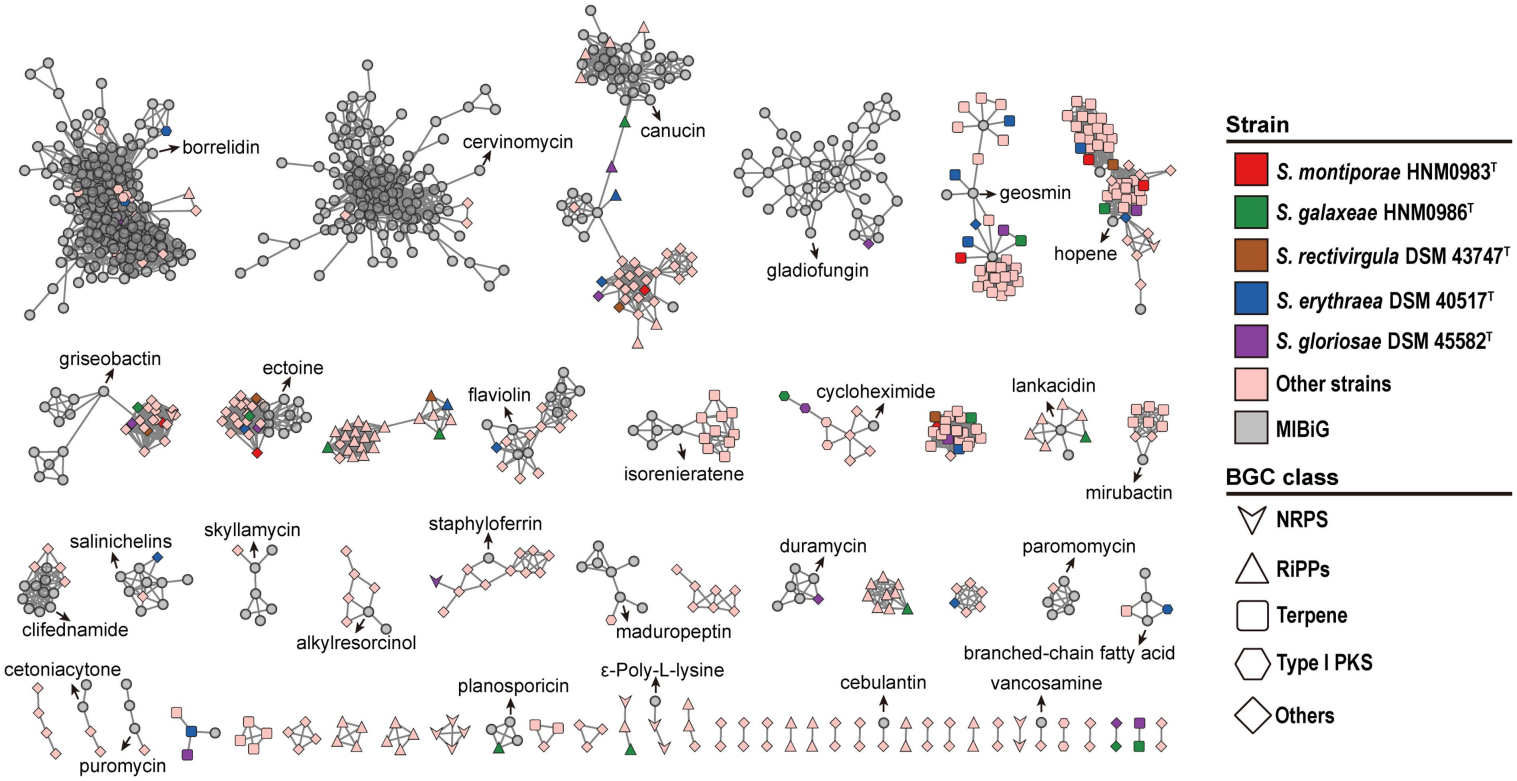
Sequence similarity network of all BGCs in 22 *Saccharopolyspora* strains. Each node represents one BGC, and the gray symbols represent reference BGCs in the MIBiG database (Terlouw et al., 2023).

The antiSMASH 7.0 analysis results showed that strain HNM0983^T^ harbored a total of 10 BGCs for the biosynthesis of secondary metabolites, while HNM0986^T^ had 26 BGCs (Supplementary Table S5). The annotation also showed that some gene clusters shared homology with BGCs of known active compounds, including cinnapeptin (Zhang and Seyedsayamdost, 2020), borregomycin (Chang and Brady, 2013), pyralomicin 1a (Kawamura et al., 1996), and ectoine (Chen et al., 2023). However, the lower gene cluster similarity indicates that these gene clusters may encode some new compounds. Furthermore, the composition of 519 BGCs from 22 strains of the genus *Saccharopolyspora* was analyzed by the BiG-SCAPE server. The results showed that terpene BGC was the most abundant in the genus *Saccharopolyspora,* and these strains widely had good terpene synthesis capacity (Figure 6). In general, marine bacteria are not the main contributors of natural terpene products, but multiple active terpenes have still been found in marine bacteria, especially in marine actinomycetes (Gozari et al., 2021). Due to the special environment of the ocean, the terpenoids from the marine bacteria may involve some special detoxification reactions, such as chlorination and bromination (Gozari et al., 2021). In addition, various types of polyketide synthase (PKS), non-ribosomal peptide synthetase (NRPS), and hybrid gene clusters were also widely distributed in *Saccharopolyspora* (Figure 6), and dozens of related products have been found in *Saccharopolyspora* (Sayed et al., 2020). A total of 395 BGCs from the *Saccharopolyspora* were found in the BGCs network. This finding showed that there remains a significant number of novel BGCs within this genus (Figure 6). Meanwhile, among the 2,735 reference BGCs in the MIBiG database (Terlouw et al., 2023), 534 of them were detected. Interestingly, the majority of the reference BGCs were only associated with a small number of BGCs from the *Saccharopolyspora* (Figure 6). The BGCs from the genus *Saccharopolyspora* tended to cluster closely together in the BGCs network, possibly indicating that these BGCs were of a conservative type in the evolution of this genus.

With the misuse of antibiotics, the problem of drug-resistant pathogens is becoming more and more serious. The pressing need for new bioactive molecules has led researchers to focus on novel microbes in extreme environments (Carroll et al., 2023). Although the genus *Saccharopolyspora* has received less attention, some active molecules from strains of this genus have been discovered and reported. The strains HNM0983^T^ and HNM0986^T^ isolated in this study further expanded the members of the genus *Saccharopolyspora* and provided new research objects for exploring metabolites from the genus *Saccharopolyspora*.

## 4 Conclusion

Marine actinomycetes associated with stony corals have been highlighted as a potential hotspot for new bioactive metabolites (Siro et al., 2022). Considering the fact that a new species contains novel BGCs, encoding the novel bioactive metabolites (Shi et al., 2022), these new *Saccharopolyspora* species isolated from stony corals in the present study show certain potential to produce novel bioactive substances for medical or agricultural use. From the combination of phylogenetic, genomic, phenotypic, and chemotaxonomic characteristics presented, we believe that two strains should be considered as novel species of the genus *Saccharopolyspora*, for which *Saccharopolyspora montiporae* sp. nov. HNM0983^T^ and *Saccharopolyspora galaxeae* sp. nov. HNM0986^T^ are proposed.

### 4.1 Description of *Saccharopolyspora montiporae* sp. nov.

*Saccharopolyspora montiporae* (mo.nti.poʼrae. N.L. gen.n. *montiporae* of the coral *Montipora foliosa* from which the type strain was isolated) is a novel Gram-stain-positive, non-motile, aerobic actinobacterium that forms well-developed and extensively branched substrate mycelium. Good growth occurs on ISP 2, moderate growth on ISP 3 and ISP 7, and poor growth on ISP 1, ISP 5, ISP 6, and Czapek agar. Growth occurs at pH 5.0–10.0 (optimum 8.0–9.0), 10–37°C (optimum 28°C), and 0–20% (w/v) NaCl (optimum 0–1%). Catalase, nitrate reductase, melanin, and H_2_S production tests are all positive, while tyrosine hydrolysis, milk coagulation, and amylase tests are all negative. D-glucose, D-fructose, D-galactose, and D-sorbitol are utilized as sole carbon sources but sucrose, raffinose, maltose, cellobiose, trehalose, L-rhamnose, L-arabinose, D-xylose, D-mannose, and D-melibiose are not. L-asparagine, L-histidine, L-arginine, L-adenine, and L-cysteine are used as sole nitrogen sources but L-serine and L-proline are not. The major menaquinone is MK-9(H_4_). Major polar lipids include phosphatidylglycerol, phosphatidylinositol, phosphatidylinositol mannosides, phosphatidylcholine, phosphatidylethanolamine, phosphatidyl-*N*-acetylglucosamine, and three unknown phospholipids. The cell-wall diamino acid is *meso*-diaminopimelic acid. Major fatty acids are *iso*-C_15:0_, *iso*-C_16:0_, *iso*-C_17:0_ and *anteiso*-C_17:0_. The type strain is HNM0983^T^ (=CCTCC AA 2020014^T^ = KCTC 49526^T^). The genome size of the strain is 4.16 Mbp, and the G + C content is 71.4%. The GenBank accession numbers for the 16S rRNA gene and the whole-genome sequence are MW131648 and JADEYC000000000, respectively.

### 4.2 Description of *Saccharopolyspora galaxeae* sp. nov.

*Saccharopolyspora galaxeae* (ga. laʼ xe. ae. N.L. gen.n. *galaxeae* of the coral *Galaxea astreata* from which the type strain was isolated) is a novel Gram-stain-positive, non-motile, aerobic actinobacterium that forms well-developed and extensively branched substrate mycelium. Good growth occurs on ISP 2, ATCC172, and Czapek agar, moderate growth on ISP 3, and poor growth on ISP 7. Growth occurs at pH 5.0–10.0 (optimum 7.0), 10–37°C (optimum 28°C), and 0–15% (w/v) NaCl (optimum 0–7%). Catalase, nitrate reductase, milk coagulation, and H_2_S production tests are all positive, while amylase, tyrosine hydrolysis, and melanin tests are all negative. Sucrose, raffinose, maltose, cellobiose, D-glucose, D-fructose, D-mannose, and D-melibiose are utilized as sole carbon sources but trehalose, L-rhamnose, L-arabinose, D-galactose, D-xylose, and D-sorbitol are not. L-histidine, L-adenine, and L-cysteine are used as sole nitrogen sources but L-asparagine, L-serine, L-arginine, and L-proline are not. The major menaquinone is MK-9(H_4_). The major polar lipids include phosphatidylglycerol, phosphatidylinositol, phosphatidylcholine phosphatidylethanolamine, and diphosphatidylglycerol. Cell-wall diamino acid is *meso*-diaminopimelic acid. The major fatty acids are *iso*-C_15:0_, *iso*-C_16:0_, and *anteiso*-C_17:0_. The type strain is HNM0986^T^ (=CCTCC AA 2020011^T^ = KCTC 49524^T^). The genome size of the strain is 6.39 Mbp, and the G + C content is 70.3%. The GenBank accession numbers for the 16S rRNA gene and the whole-genome sequence are MW488440 and JADDUE000000000, respectively.

## Data Availability

The datasets presented in this study can be found in online repositories. The names of the repository/repositories and accession number(s) can be found at: JADEYC000000000 JADDUE000000000.

## Footnotes

^1^ https://lpsn.dsmz.de/genus/saccharopolyspora
