# Supplementary material for: Comprehensive genome analysis of two novel Saccharopolyspora strains—Saccharopolyspora montiporae sp. nov. and Saccharopolyspora galaxeae sp. nov. isolated from stony corals in Hainan
Source: Front Microbiol. 2024 Nov 13;15:1432042. doi: 10.3389/fmicb.2024.1432042 (PMC11599206; doi:10.3389/fmicb.2024.1432042)
Supplement: Supplementary file 1 [file Data_Sheet_1.docx]

Supplementary Material

**Comprehensive genome analysis of two novel *Saccharopolyspora* strains—*Saccharopolyspora montiporae* sp. nov. and *Saccharopolyspora galaxeae* sp. nov. isolated from stony corals in Hainan**

**Yuhui Xie^1,2†^, Fenfa Li^1†^, Qingyi Xie^3^, Fandong Kong^3^, Yun Xu^1^, Qingyun Ma^3^, Wenqiang Wu^1^, Dongyi Huang^1^, Xinqiang Xie^4^, Shuangqing Zhou^1,5*^, Youxing Zhao^3*^ and Xiaolong Huang^1*^**

^1^School of Life and Health Sciences, Hainan University, Haikou, China.

^2^Frontiers Science Center for Synthetic Biology and Key Laboratory of Systems Bioengineering (Ministry of Education), School of Chemical Engineering and Technology, Tianjin University, Tianjin, 300072, China.

^3^Haikou Key Laboratory for Research and Utilization of Tropical Natural Products, Institute of Tropical Bioscience and Biotechnology, Chinese Academy of Tropical Agricultural Sciences, Haikou, China.

^4^Guangdong Institute of Microbiology, Guangdong Academy of Sciences, Guangzhou, China.

^5^College of Pharmacy, Guilin Medical University, Guilin, China.

†These authors contributed equally to this work.

*** Correspondence:**

Xiaolong Huang, E-mail: huangxialong@hainanu.edu.cn;

Shuangqing Zhou, [zsq2020@glmc.edu.cn;](mailto:zsq2020@plmc.edu.cn;)

Youxing Zhao, E-mail: [zhaoyouxing@itbb.org.cn](mailto:zhaoyouxing@itbb.org.cn)

Supplemental material Content

**Figure S1.** Maximum-likelihood phylogenetic tree derived by 16S rRNA sequences.

**Figure S2.** Maximum-parsimony phylogenetic tree derived by 16S rRNA sequences.

**Figure S3.** The phospholipid profile of strain HNM0983 ^T^.

**Figure S4.** The phospholipid profile of strain HNM0986 ^T^.

**Figure S5.** Genome quality information and genome circle map of strains HNM0983^T^ and HNM0986^T^.

**Table S1.** The dDDH and ANI values analysis result of *S. montiporae* HNM0983^T^, *S. galaxeae* HNM0986^T^ and other type strains of the genus *Saccharopolyspora*.

**Table S2.** Cultural characteristics of strains HNM0983^T^ and HNM0986^T^.

**Table S3.** Annotation of genes associated with habitat adaptation in strain HNM0983^T^.

**Table S4.** Annotation of genes associated with habitat adaptation in strain HNM0986^T^.

**Table S5.** Annotation results of biosynthetic gene clusters of strains HNM0983^T^ and HNM0986^T^.

## Supplementary Figures

**Supplementary Figure 1.** Maximum-likelihood phylogenetic tree derived by 16S rRNA sequences, showing the relationships between HNM0983^T^, HNM0986^T^ and other type strains of genus *Saccharopolyspora*. Only values above 50% were shown. Bar 5 nucleotide substitutions per 1000 nucleotides.


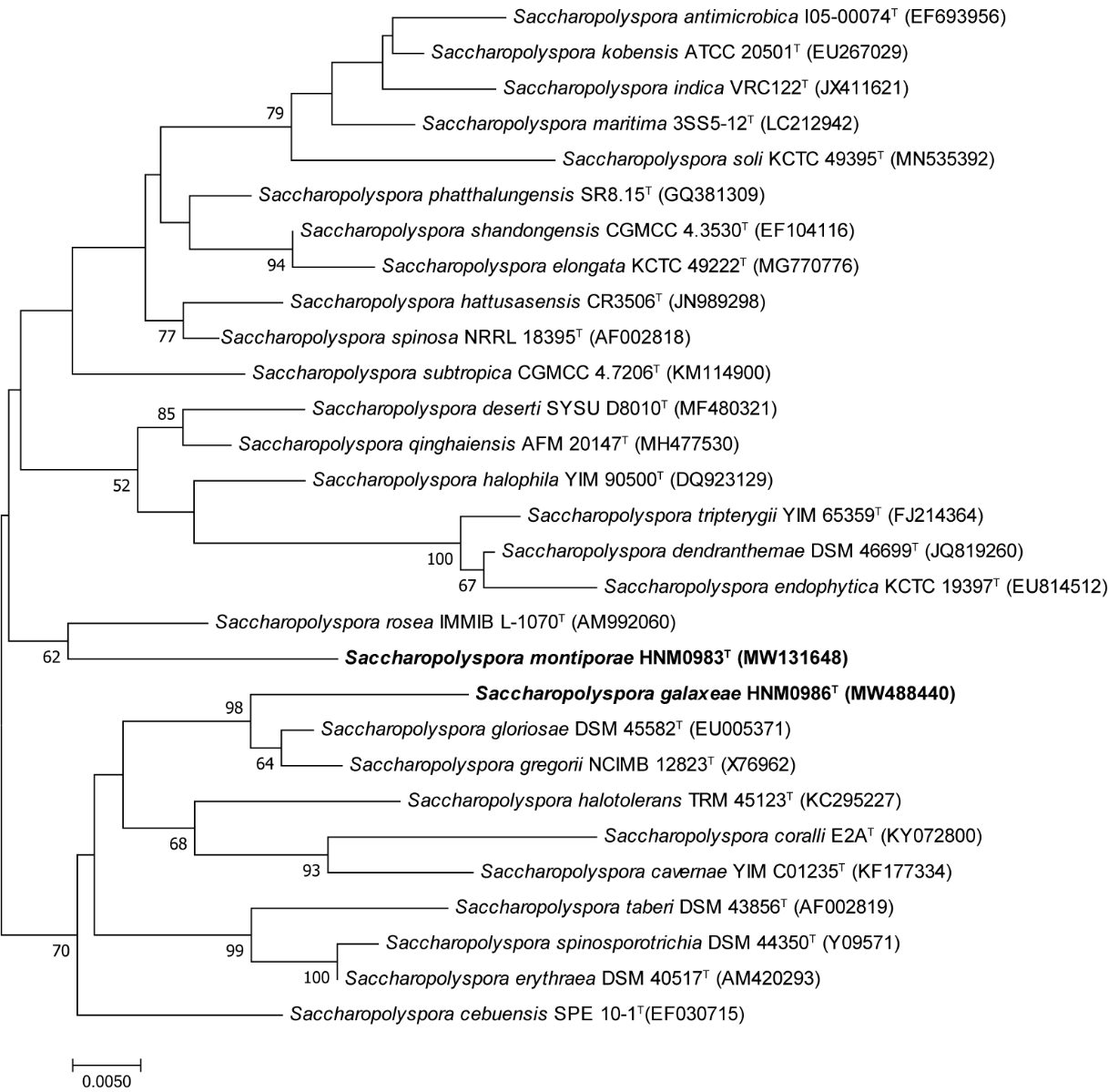


**Supplementary Figure 2.** Maximum-parsimony phylogenetic tree derived by 16S rRNA sequences, showing the relationships between HNM0983^T^, HNM0986^T^ and other type strains of genus *Saccharopolyspora*. Only values above 50% were shown.


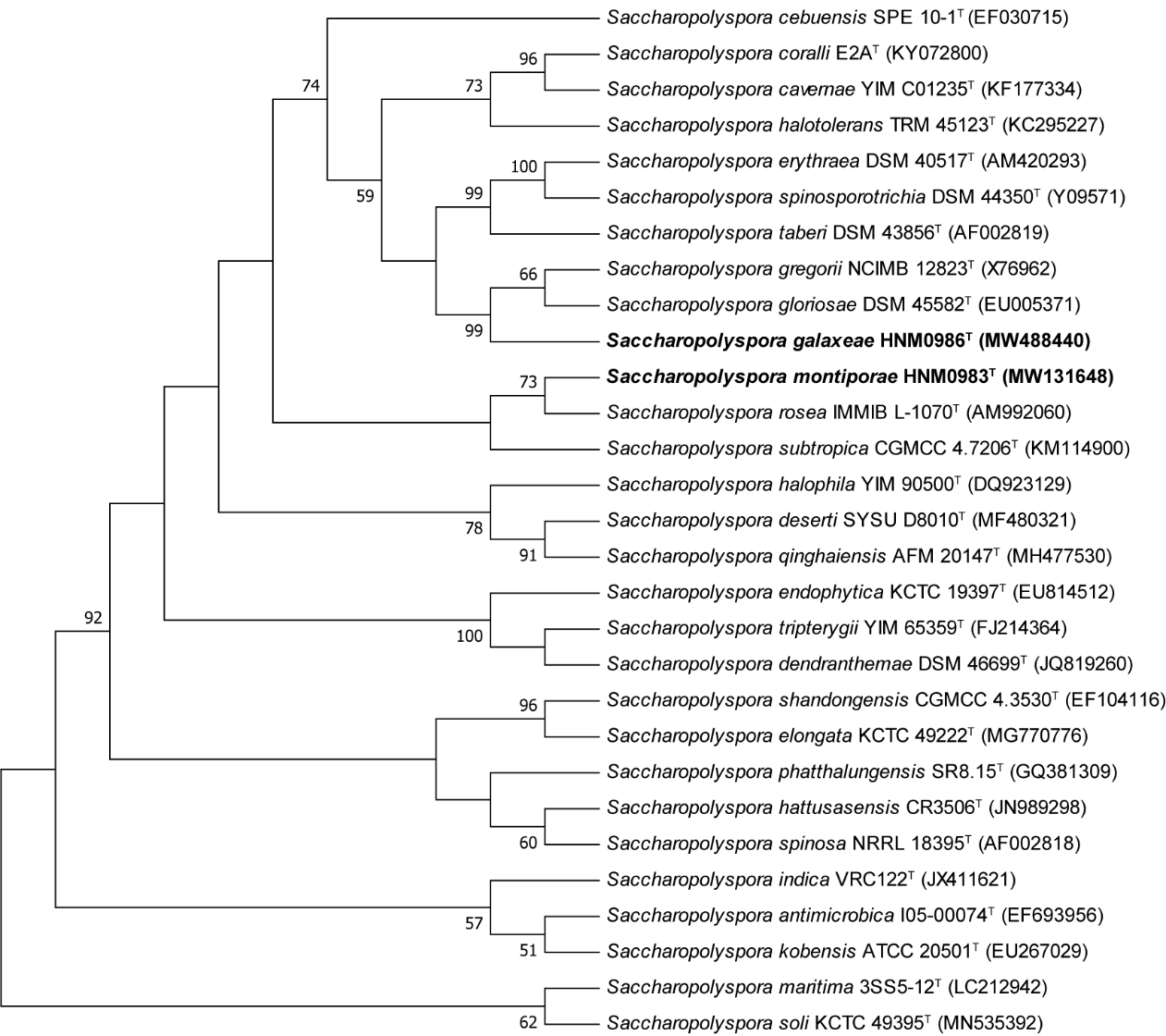


**Supplementary Figure 3.** The phospholipid profile of HNM0983^T^.

Chloroform-methanol-water (65:25:4) was used in the first direction, followed by chloroform-acetic acid-methanol-water (80:15:12:4) in the second direction.

Abbreviations: PG, phosphatidylglycerol; PI, phosphatidylinositol; PIM, phosphatidylinositol mannosides; PC, phosphatidylcholine; PE, phosphatidylethanolamine; PL, unknown phospholipid; NPG, phosphatidyl-N-acetylglucosamine.


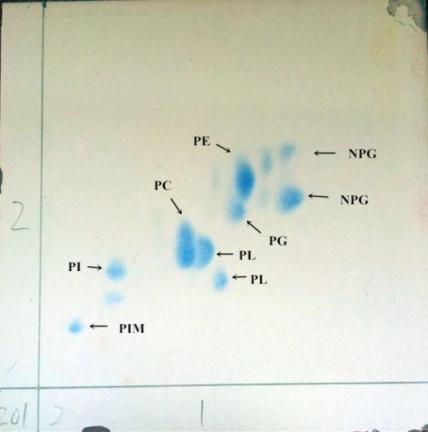

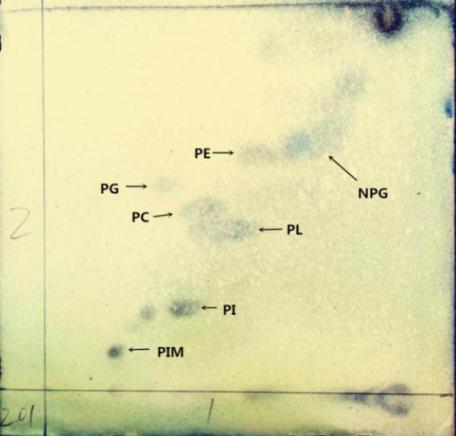

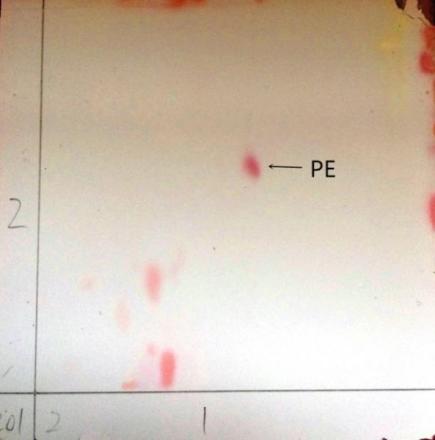


(a). Dyed by molybdenum blue reagent. **(b).** Dyed by phosphomolybdic acid reagent. (C) Dyed by ninhydrin reagent

**Supplementary Figure 4.** The phospholipid profile of HNM0986^T^.

Chloroform-methanol-water (65:25:4) was used in the first direction, followed by chloroform-acetic acid-methanol-water (80:15:12:4) in the second direction.

Abbreviations: PG, phosphatidylglycerol; PI, phosphatidylinositol; PC, phosphatidylcholine; PE, phosphatidylethanolamine; DPG, diphosphatidylglycerol.


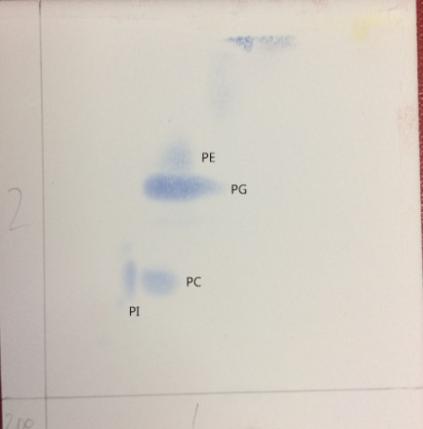

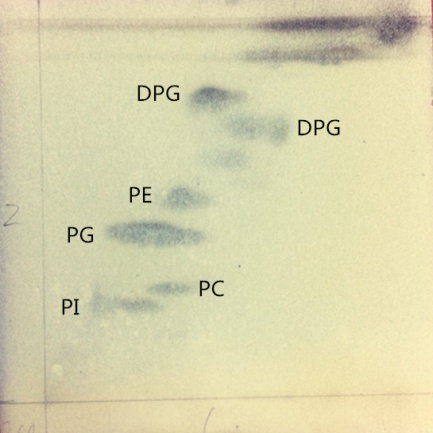

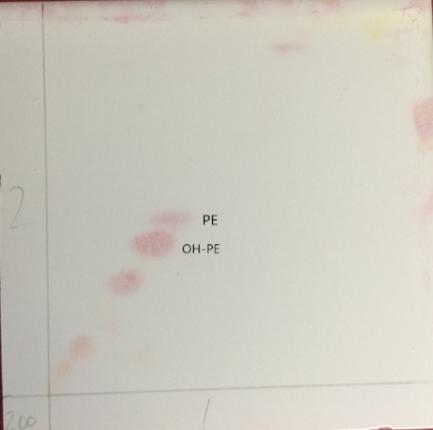


(a) Dyed by molybdenum blue reagent. **(b)** Dyed by phosphomolybdic acid reagent. (C) Dyed by ninhydrin reagent

**Supplementary Figure 5.** Genome quality information and genome circle map of strains HNM0983^T^ and HNM0986^T^.


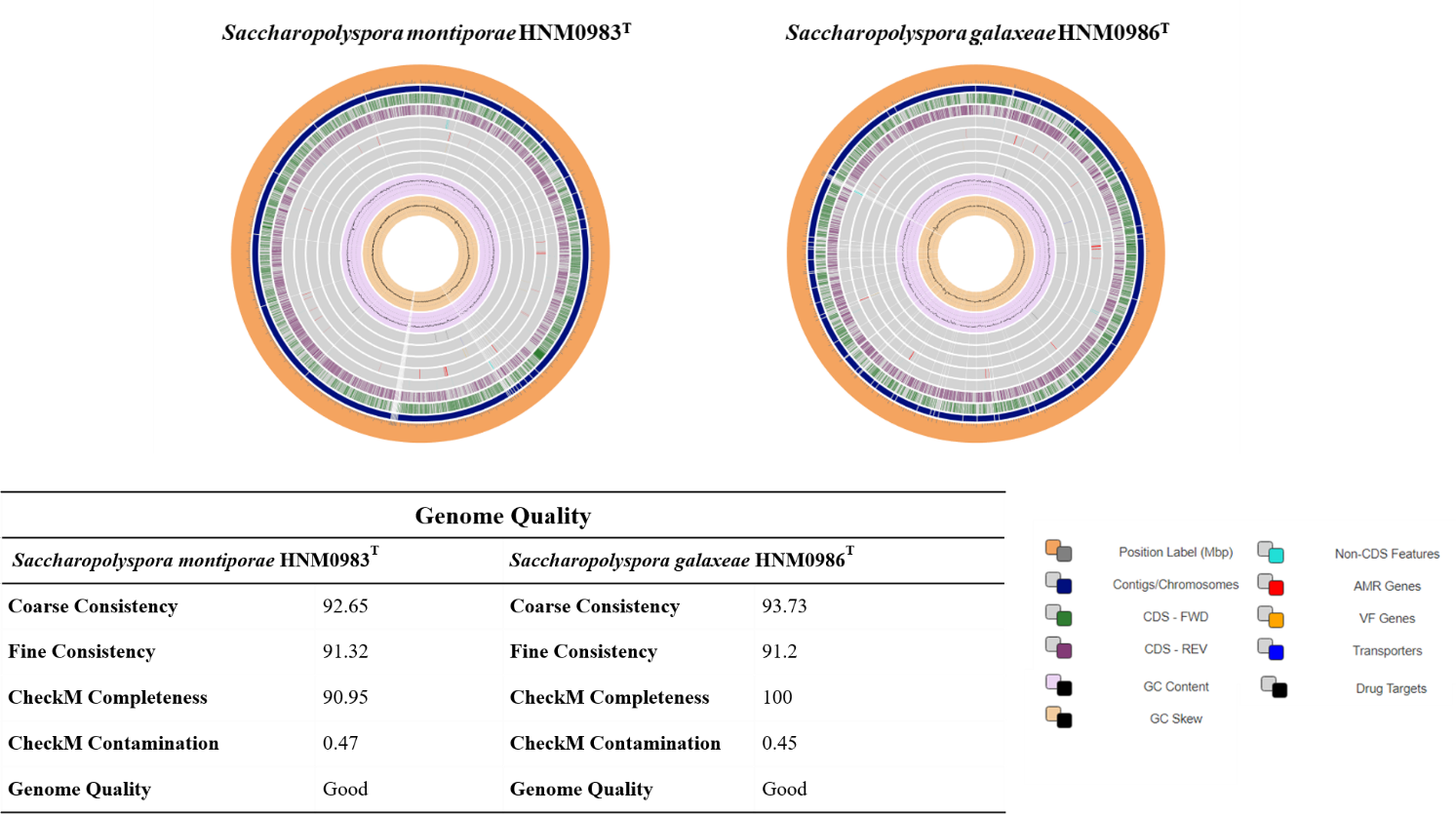


## Supplementary Tables

**Supplementary Table 1.** The dDDH and ANI values analysis result of *S. montiporae* HNM0983^T^, *S. galaxeae* HNM0986^T^ and other type strains of the genus *Saccharopolyspora*.

| Strain | dDDH (%) | | ANI (%) | |
| --- | --- | --- | --- | --- |
|  | HNM0983^T^ | HNM0986^T^ | HNM0983^T^ | HNM0986^T^ |
| *S. montiporae* HNM0983^T^ | - | 21.4 | - | 77.2 |
| *S. galaxeae* HNM0986^T^ | 21.4 | - | 77.3 | - |
| *S. antimicrobica* DSM 45119^T^ | 22.8 | 22.8 | 78.8 | 78.2 |
| *S. aridisoli* 16K404^T^ | 21.1 | 21.3 | 77.2 | 77.0 |
| *S. dendranthemae* DSM 46699^T^ | 21.0 | 21.2 | 77.2 | 76.9 |
| *S. elongata* 7K502^T^ | 22.5 | 22.4 | 78.7 | 78.6 |
| *S. endophytica* KCTC 19397^T^ | 21.3 | 21.8 | 77.7 | 77.6 |
| *S. erythraea* DSM 40517^T^ | 22.0 | 22.8 | 78.1 | 78.5 |
| *S. flava* DSM 44771^T^ | 21.3 | 21.7 | 78.0 | 77.5 |
| *S. gloriosae* DSM 45582^T^ | 21.4 | 24.6 | 77.4 | 81.3 |
| *S. hirsuta* VKM Ac-666^T^ | 22.5 | 22.5 | 79.0 | 78.5 |
| *S. hordei* DSM 44065^T^ | 22.1 | 22.4 | 78.8 | 78.5 |
| *S. karakumensis* 5K548^T^ | 21.3 | 21.5 | 77.4 | 77.3 |
| *S. kobensis* ATCC 20501^T^ | 22.6 | 22.5 | 78.8 | 78.3 |
| *S. phatthalungensis* DSM 45584^T^ | 22.3 | 22.0 | 77.8 | 77.6 |
| *S. rectivirgula* DSM 43747^T^ | 20.4 | 21.2 | 76.7 | 76.6 |
| *S. rhizosphaerae* H219^T^ | 21.1 | 21.7 | 77.2 | 77.2 |
| *S. shandongensis* CGMCC 4.3530^T^ | 22.4 | 22.4 | 78.7 | 78.3 |
| *S. soli* K220^T^ | 22.2 | 22.0 | 78.1 | 77.6 |
| *S. spinosa* DSM 44228^T^ | 22.5 | 22.6 | 78.3 | 77.9 |
| *S. subtropica* CGMCC 4.7206^T^ | 22.2 | 22.3 | 78.6 | 78.6 |
| *S.terrae* 16K309^T^ | 21.2 | 21.5 | 77.6 | 77.4 |

**Supplementary Table 2.** Cultural characteristics of strains HNM0983^T^ and HNM0986^T^.

Abbreviations: A, Abundant; D, does not grow; LY, Light yellow; M, Moderate; P, Poor; W, White; Y, yellow.

| Medium | HNM0983^T^/ HNM0986^T^ | | |
| --- | --- | --- | --- |
|  | Growth | Aerial mycelium | Substrate mycelium |
| Tryptone-yeast extract broth (ISP 1) | P/ D | W/ D | W/ D |
| Yeast extract/malt extract agar (ISP 2) | A/A | W/ Y | LY/ LY |
| Oatmeal agar (ISP 3) | M/M | W/ W | W/ W |
| Inorganic salts/starch agar (ISP 4) | D/ D | D/ D | D/ D |
| Glycerol-asparagine agar (ISP 5) | P/ D | W/ D | W/ D |
| Peptone-yeast extract iron agar (ISP 6) | P/ D | W/ D | W/ D |
| Tyrosine agar (ISP 7) | M/ P | W/ W | W/ W |
| Czapek agar  ATCC172 | P/ A  A/ A | W/ Y  W/ W | LY/ Y  W/ LY |

**Supplementary Table 3.** Annotation of genes associated with habitat adaptation in strain HNM0983^T^.

| Start | End | AA Length | Annotation |
| --- | --- | --- | --- |
| **Cold shock protein** | | | |
| 167693 | 168100 | 135 | Cold shock protein of CSP family |
| 217677 | 217880 | 67 | Cold shock protein of CSP family |
| **Heat shock protein** | | | |
| 107653 | 107943 | 96 | Heat shock protein 10 kDa family chaperone GroES |
| 214259 | 215896 | 545 | Heat shock protein 60 kDa family chaperone GroEL |
| 108063 | 109682 | 539 | Heat shock protein 60 kDa family chaperone GroEL |
| 11824 | 13473 | 549 | Heat shock protein 60 kDa family chaperone GroEL |
| 51481 | 52197 | 238 | Heat shock protein GrpE |
| **Betaine** | | | |
| 251326 | 252315 | 329 | Glycine betaine ABC transport system, ATP-binding protein OpuAA (EC 3.6.3.32) |
| 1 | 318 | 106 | Glycine betaine ABC transport system, ATP-binding protein OpuAA (EC 3.6.3.32) |
| 80539 | 81723 | 394 | Glycine betaine ABC transport system, ATP-binding protein OpuAA (EC 3.6.3.32) |
| 249480 | 250382 | 300 | Glycine betaine ABC transport system, glycine betaine-binding protein OpuAC |
| 81868 | 82806 | 312 | Glycine betaine ABC transport system, glycine betaine-binding protein OpuAC |
| 250434 | 251333 | 299 | Glycine betaine ABC transport system, permease protein OpuAB |
| 79635 | 80546 | 303 | Glycine betaine ABC transport system, permease protein OpuAB |
| 145778 | 147271 | 497 | L-Proline/Glycine betaine transporter ProP |
| 9273 | 10691 | 472 | L-Proline/Glycine betaine transporter ProP |
| 143852 | 145231 | 459 | L-Proline/Glycine betaine transporter ProP |
| 148827 | 150419 | 530 | Glycine betaine transporter OpuD |
| 22256 | 23992 | 578 | Glycine betaine transporter OpuD |
| 57502 | 59397 | 631 | Glycine betaine transporter OpuD |
| 13354 | 14637 | 427 | Proline/betaine transporter |
| **Na^+^** | | | |
| 226401 | 228782 | 793 | Na(^+^) H(^+^) antiporter subunit A |
| 228779 | 229324 | 181 | Na(^+^) H(^+^) antiporter subunit B |
| 229321 | 229737 | 138 | Na(^+^) H(^+^) antiporter subunit C |
| 229734 | 231269 | 511 | Na(^+^) H(^+^) antiporter subunit D |
| 231262 | 231813 | 183 | Na(^+^) H(^+^) antiporter subunit E |
| 231866 | 232132 | 88 | Na(^+^) H(^+^) antiporter subunit F |
| 232129 | 232479 | 116 | Na(^+^) H(^+^) antiporter subunit G |
| 118225 | 119742 | 505 | Na(^+^)-dependent bicarbonate transporter BicA |
| 60357 | 61949 | 530 | Na(^+^)-linked D-alanine glycine permease |
| 61709 | 63013 | 434 | Na(^+^) dependent nucleoside transporter NupC |
| 76907 | 78520 | 537 | Na^+^/H^+^ antiporter |
| 23053 | 24294 | 413 | Na+/H+ antiporter NhaA type |
| 41835 | 43226 | 463 | Na+/H+-dicarboxylate symporter |
| 74587 | 75933 | 448 | Na+/H+-dicarboxylate symporter |
| 42928 | 44400 | 490 | Na+/solute symporter |
| **Superoxide dismutase** | | | |
| 38663 | 39070 | 135 | Nickel-dependent superoxide dismutase (EC 1.15.1.1) |
| 157641 | 158270 | 209 | Superoxide dismutase [Mn/Fe] (EC 1.15.1.1) |
| **Catalase** | | | |
| 112702 | 114183 | 493 | Catalase KatE (EC 1.11.1.6) |
| 9987 | 11438 | 483 | Catalase KatE (EC 1.11.1.6) |
| 105 | 2315 | 736 | Catalase-peroxidase KatG (EC 1.11.1.21) |
| **Heavy metal antagonism, transport** | | | |
| 148970 | 149377 | 135 | Arsenate reductase (EC 1.20.4.4) thioredoxin-coupled, LMWP family |
| 150476 | 150793 | 105 | Arsenical resistance operon repressor |
| 149391 | 150479 | 362 | Arsenical-resistance protein ACR3 |
| 21837 | 23180 | 447 | Arsenite/antimonite:H+ antiporter ArsB |
| 58603 | 60837 | 744 | Lead, cadmium, zinc and mercury transporting ATPase (EC 3.6.3.3) (EC 3.6.3.5); Copper-translocating P-type ATPase (EC 3.6.3.4) |
| 108658 | 110643 | 661 | Lead, cadmium, zinc and mercury transporting ATPase (EC 3.6.3.3) (EC 3.6.3.5); Copper-translocating P-type ATPase (EC 3.6.3.4) |
| 74879 | 76921 | 680 | Lead, cadmium, zinc and mercury transporting ATPase (EC 3.6.3.3) (EC 3.6.3.5); Copper-translocating P-type ATPase (EC 3.6.3.4) |
| 11351 | 13324 | 657 | Lead, cadmium, zinc and mercury transporting ATPase (EC 3.6.3.3) (EC 3.6.3.5); Copper-translocating P-type ATPase (EC 3.6.3.4) |
| 66600 | 69071 | 823 | Lead, cadmium, zinc and mercury transporting ATPase (EC 3.6.3.3) (EC 3.6.3.5); Copper-translocating P-type ATPase (EC 3.6.3.4) |
| 172334 | 173107 | 257 | Cobalt ABC transporter, ATP-binding protein CbtL |
| 171306 | 172337 | 343 | Cobalt ABC transporter, permease protein CbtK |
| 170269 | 171309 | 346 | Cobalt ABC transporter, substrate-binding protein CbtJ |
| 29276 | 30193 | 305 | Cobalt/zinc/cadmium resistance protein CzcD |
| 103610 | 104932 | 440 | Magnesium and cobalt efflux protein CorC |
| 79572 | 80327 | 251 | Manganese ABC transporter, ATP-binding protein SitB |
| 80324 | 81220 | 298 | Manganese ABC transporter, inner membrane permease protein SitC @, inner membrane permease protein SitD |
| 78610 | 79575 | 321 | Manganese ABC transporter, periplasmic-binding protein SitA |
| 36779 | 38071 | 430 | Manganese transport protein MntH |
| 113734 | 114147 | 137 | Zinc uptake regulation protein Zur |
| 261891 | 262703 | 270 | Zinc ABC transporter, ATP-binding protein ZnuC |
| 260878 | 261864 | 328 | Zinc ABC transporter, periplasmic-binding protein ZnuA |
| 262700 | 263575 | 291 | Zinc ABC transporter, permease protein ZnuB |
| 238648 | 240240 | 530 | ABC transporter, permease protein (nickel/peptides/opines) |
| 123087 | 124781 | 564 | ABC transporter, permease protein (nickel/peptides/opines) |
| 94041 | 95489 | 482 | ABC transporter, permease protein (nickel/peptides/opines) |
| 57655 | 59412 | 585 | ABC transporter, permease protein (nickel/peptides/opines) |
| 236745 | 237779 | 344 | ABC transporter, permease protein (nickel/peptides/opines) |
| 96327 | 97340 | 337 | ABC transporter, permease protein (nickel/peptides/opines) |
| 60369 | 61322 | 317 | ABC transporter, permease protein (nickel/peptides/opines) |
| 187003 | 188736 | 577 | ABC transporter, permease protein (nickel/peptides/opines) |
| 8974 | 9915 | 313 | ABC transporter, permease protein (nickel/peptides/opines) |
| 95479 | 96330 | 283 | ABC transporter, permease protein (nickel/peptides/opines) |
| 185513 | 187006 | 497 | ABC transporter, permease protein (nickel/peptides/opines) |
| 98140 | 99687 | 515 | ABC transporter, permease protein (nickel/peptides/opines) |
| 12265 | 13797 | 510 | ABC transporter, permease protein (nickel/peptides/opines) |
| 61336 | 62925 | 529 | ABC transporter, permease protein (nickel/peptides/opines) |
| 67377 | 69134 | 585 | ABC transporter, permease protein (nickel/peptides/opines) |

**Supplementary Table 4.** Annotation of genes associated with habitat adaptation in strain HNM0986^T^.

| Start | End | AA Length | Annotation |
| --- | --- | --- | --- |
| **Cold shock protein** | | | |
| 579915 | 580118 | 67 | Cold shock protein of CSP family |
| 166563 | 166766 | 67 | Cold shock protein of CSP family |
| 434481 | 434684 | 67 | Cold shock protein of CSP family |
| 161697 | 161900 | 67 | Cold shock protein of CSP family |
| 9116 | 9319 | 67 | Cold shock protein of CSP family |
| 633789 | 634190 | 133 | Cold shock protein of CSP family => SCO4325 |
| **Heat shock protein** | | | |
| 736721 | 737011 | 96 | Heat shock protein 10 kDa family chaperone GroES |
| 582090 | 583727 | 545 | Heat shock protein 60 kDa family chaperone GroEL |
| 737260 | 738876 | 538 | Heat shock protein 60 kDa family chaperone GroEL |
| 4632 | 5384 | 250 | Heat shock protein GrpE |
| 341975 | 342565 | 196 | Heat shock protein GrpE |
| 265310 | 265804 | 164 | HEAT SHOCK PROTEIN HSP |
| **Betaine** | | | |
| 372684 | 373871 | 395 | Glycine betaine ABC transport system, ATP-binding protein OpuAA (EC 3.6.3.32) |
| 374751 | 375653 | 300 | Glycine betaine ABC transport system, glycine betaine-binding protein OpuAC |
| 373864 | 374733 | 289 | Glycine betaine ABC transport system, permease protein OpuAB |
| 197406 | 198401 | 331 | glycine betaine ABC transporter substrate-binding protein |
| 192950 | 194734 | 594 | Glycine betaine transporter OpuD |
| 181850 | 183283 | 477 | L-Proline/Glycine betaine transporter ProP |
| 346246 | 347592 | 448 | L-Proline/Glycine betaine transporter ProP |
| 267573 | 269006 | 477 | L-Proline/Glycine betaine transporter ProP |
| 54164 | 55597 | 477 | L-Proline/Glycine betaine transporter ProP |
| 93361 | 95004 | 547 | Betaine/carnitine/choline transporter (BCCT) family |
| **Na^+^** | | | |
| 963948 | 966320 | 790 | Na(^+^) H(^+^) antiporter subunit A |
| 966317 | 966874 | 185 | Na(^+^) H(^+^) antiporter subunit B |
| 966871 | 967299 | 142 | Na(^+^) H(^+^) antiporter subunit C |
| 967296 | 968828 | 510 | Na(^+^) H(^+^) antiporter subunit D |
| 968821 | 969375 | 184 | Na(^+^) H(^+^) antiporter subunit E |
| 969375 | 969641 | 88 | Na(^+^) H(^+^) antiporter subunit F |
| 969638 | 969985 | 115 | Na(^+^) H(^+^) antiporter subunit G |
| 394526 | 396022 | 498 | Na(^+^)/H(^+^) antiporter |
| 513796 | 515310 | 504 | Na(^+^)-dependent bicarbonate transporter BicA |
| 102264 | 103658 | 464 | Na^+^ dependent nucleoside transporter NupC |
| 88304 | 89860 | 518 | Na^+^/H^+^ antiporter |
| 13615 | 14397 | 260 | Na^+^/H^+^ antiporter |
| 430962 | 432179 | 405 | Na^+^/H^+^ antiporter NhaA type |
| 810630 | 812444 | 604 | Na^+^/H^+^ antiporter NhaA type |
| 352449 | 352724 | 91 | Na^+^/H^+^ antiporter NhaA type |
| 69913 | 71295 | 460 | Na^+^/H^+^-dicarboxylate symporter |
| 43128 | 44465 | 445 | Na^+^/H^+^-dicarboxylate symporter |
| **Superoxide dismutase** | | | |
| 92189 | 92596 | 135 | Nickel-dependent superoxide dismutase (EC 1.15.1.1) |
| 647474 | 648103 | 209 | Superoxide dismutase [Mn/Fe] (EC 1.15.1.1) |
| **Catalase** | | | |
| 388324 | 389775 | 483 | Catalase KatE (EC 1.11.1.6) |
| 424281 | 426401 | 706 | Catalase KatE-intracellular protease (EC 1.11.1.6) |
| 342793 | 345021 | 742 | Catalase-peroxidase KatG (EC 1.11.1.21) |
| 253092 | 255287 | 731 | Catalase-peroxidase KatG (EC 1.11.1.21) |
| **Heavy metal antagonism, transport** | | | |
| 148970 | 149377 | 135 | Arsenate reductase (EC 1.20.4.4) thioredoxin-coupled, LMWP family |
| 150476 | 150793 | 105 | Arsenical resistance operon repressor |
| 149391 | 150479 | 362 | Arsenical-resistance protein ACR3 |
| 317340 | 317765 | 141 | Arsenate reductase (EC 1.20.4.4) thioredoxin-coupled, LMWP family |
| 587613 | 588287 | 224 | Arsenate reductase (EC 1.20.4.4) thioredoxin-coupled, LMWP family |
| 321519 | 322196 | 225 | Arsenate reductase (EC 1.20.4.4) thioredoxin-coupled, LMWP family |
| 318862 | 319233 | 123 | Arsenical resistance operon repressor |
| 588388 | 588669 | 93 | Arsenical resistance operon repressor |
| 321213 | 321416 | 67 | Arsenical resistance operon repressor |
| 317762 | 318865 | 367 | Arsenical-resistance protein ACR3 |
| 589338 | 591137 | 599 | Arsenite/antimonite pump-driving ATPase ArsA (EC 3.6.3.16) |
| 318598 | 320472 | 624 | Arsenite/antimonite pump-driving ATPase ArsA (EC 3.6.3.16) |
| 591207 | 592673 | 488 | Arsenite/antimonite:H+ antiporter ArsB |
| 317135 | 318601 | 488 | Arsenite/antimonite:H+ antiporter ArsB |
| 77912 | 79297 | 461 | Arsenite/antimonite:H+ antiporter ArsB |
| 434966 | 437263 | 765 | Lead, cadmium, zinc and mercury transporting ATPase (EC 3.6.3.3) (EC 3.6.3.5); Copper-translocating P-type ATPase (EC 3.6.3.4) |
| 23089 | 25314 | 741 | Lead, cadmium, zinc and mercury transporting ATPase (EC 3.6.3.3) (EC 3.6.3.5); Copper-translocating P-type ATPase (EC 3.6.3.4) |
| 268506 | 270467 | 653 | Lead, cadmium, zinc and mercury transporting ATPase (EC 3.6.3.3) (EC 3.6.3.5); Copper-translocating P-type ATPase (EC 3.6.3.4) |
| 301868 | 304297 | 809 | Lead, cadmium, zinc and mercury transporting ATPase (EC 3.6.3.3) (EC 3.6.3.5); Copper-translocating P-type ATPase (EC 3.6.3.4) |
| 356457 | 358790 | 777 | Lead, cadmium, zinc and mercury transporting ATPase (EC 3.6.3.3) (EC 3.6.3.5); Copper-translocating P-type ATPase (EC 3.6.3.4) |
| 71635 | 73530 | 631 | Lead, cadmium, zinc and mercury transporting ATPase (EC 3.6.3.3) (EC 3.6.3.5); Copper-translocating P-type ATPase (EC 3.6.3.4) |
| 229122 | 229901 | 259 | Cobalt ABC transporter, ATP-binding protein CbtL |
| 228103 | 229125 | 340 | Cobalt ABC transporter, permease protein CbtK |
| 226952 | 228106 | 384 | Cobalt ABC transporter, substrate-binding protein CbtJ |
| 498476 | 499408 | 310 | Cobalt/zinc/cadmium resistance protein CzcD |
| 342396 | 343460 | 354 | Cobalt-zinc-cadmium resistance protein |
| 390850 | 391893 | 347 | Magnesium and cobalt efflux protein CorC |
| 209149 | 210513 | 454 | Magnesium and cobalt efflux protein CorC |
| 345438 | 346478 | 346 | Magnesium and cobalt efflux protein CorC |
| 29325 | 30440 | 371 | Magnesium and cobalt transport protein CorA |
| 65173 | 66252 | 359 | Magnesium and cobalt transport protein CorA |
| 265597 | 266319 | 240 | Manganese ABC transporter, ATP-binding protein SitB |
| 266316 | 267188 | 290 | Manganese ABC transporter, inner membrane permease protein SitC @, inner membrane permease protein SitD |
| 264650 | 265600 | 316 | Manganese ABC transporter, periplasmic-binding protein SitA |
| 620131 | 621486 | 451 | Manganese transport protein MntH |
| 105300 | 106709 | 469 | Manganese transport protein MntH |
| 218892 | 219314 | 140 | Zinc uptake regulation protein Zur |
| 523658 | 524503 | 281 | Zinc ABC transporter, ATP-binding protein ZnuC |
| 522780 | 523658 | 292 | Zinc ABC transporter, permease protein ZnuB |
| 524547 | 525560 | 337 | Zinc ABC transporter, substrate-binding protein ZnuA |
| 649438 | 651054 | 538 | ABC transporter, substrate-binding protein (nickel/peptides/opines) |
| 94371 | 95891 | 506 | ABC transporter, substrate-binding protein (nickel/peptides/opines) |
| 10405 | 12021 | 538 | ABC transporter, substrate-binding protein (nickel/peptides/opines) |
| 29272 | 31002 | 576 | ABC transporter, substrate-binding protein (nickel/peptides/opines) |
| 390032 | 391090 | 352 | ABC transporter, permease protein (nickel/peptides/opines) |
| 236927 | 237862 | 311 | ABC transporter, permease protein (nickel/peptides/opines) |
| 95921 | 96853 | 310 | ABC transporter, permease protein (nickel/peptides/opines) |
| 9461 | 10408 | 315 | ABC transporter, permease protein (nickel/peptides/opines) |
| 96495 | 97445 | 316 | ABC transporter, permease protein (nickel/peptides/opines) |
| 31072 | 31893 | 273 | ABC transporter, permease protein (nickel/peptides/opines) |
| 652048 | 653058 | 336 | ABC transporter, permease protein (nickel/peptides/opines) |
| 8673 | 9464 | 263 | ABC transporter, permease protein (nickel/peptides/opines) |

**Supplementary Table 5.** Annotation results of biosynthetic gene clusters of strains HNM0983^T^ and HNM0986^T^.

Abbreviations: -, no data.

| HNM0983^T^ | | | | HNM0986^T^ | | | |
| --- | --- | --- | --- | --- | --- | --- | --- |
| Region | Type | Most similar known cluster | Similarity | Region | Type | Most similar known cluster | Similarity |
| Region 1 | NRP-metallophore, NRPS | cinnapeptin | 10% | Region 1 | ranthipeptide | - |  |
| Region 2 | terpene | hopene | 46% | Region 2 | hglE-KS | - |  |
| Region 3 | T1PKS | - |  | Region 3 | amglyccycl | cetoniacytone A | 22% |
| Region 4 | terpene | rifamorpholine | 6% | Region 4 | redox-cofactor | - |  |
| Region 5 | redox-cofactor | - |  | Region 5 | redox-cofactor | lankacidin C | 13% |
| Region 6 | indole | fortimicin | 6% | Region 6 | lanthipeptide-class |  |  |
| Region 7 | T3PKS | loseolamycin | 16% | Region 7 | NRPS-like | frankiamicin | 14% |
| Region 8 | NI-siderophore, terpene | 5-dimethylallylindole-3-acetonitrile | 77% | Region 8 | NI-siderophore | schizokinen | 20% |
| Region 9 | terpene | schizokinen | 20% | Region 9 | lassopeptide | anantin C | 75% |
| Region 10 | ectoine | ectoine | 75% | Region 10 | ectoine | ectoine | 100% |
|  |  |  |  | Region 11 | lanthipeptide-class | planosporicin | 60% |
|  |  |  |  | Region 12 | betalactone | reveromycin A | 6% |
|  |  |  |  | Region 13 | RiPP-like | - |  |
|  |  |  |  | Region 14 | betalactone | atolypene A/atolypene B | 11% |
|  |  |  |  | Region 15 | terpene | hopene | 38% |
|  |  |  |  | Region 16 | terpene | geosmin | 100% |
|  |  |  |  | Region 17 | terpene | SF2575 | 6% |
|  |  |  |  | Region 18 | T1PKS | kanglemycin | 6% |
|  |  |  |  | Region 19 | indole | borregomycin | 22% |
|  |  |  |  | Region 20 | RiPP-like | deoxyhangtaimycin | 2% |
|  |  |  |  | Region 21 | terpene | isorenieratene | 57% |
|  |  |  |  | Region 22 | NRP-metallophore, NRPS | marformycin | 16% |
|  |  |  |  | Region 23 | CDPS | - |  |
|  |  |  |  | Region 24 | arylpolyene | oviedomycin | 8% |
|  |  |  |  | Region 25 | redox-cofactor | lankacidin C | 26% |
|  |  |  |  | Region 26 | amglyccycl | pyralomicin1a | 18% |
